# INSM1 Expression in Breast Neoplasms with Neuroedocrine Features

**DOI:** 10.1007/s12022-021-09682-1

**Published:** 2021-05-19

**Authors:** Jasna Metovic, Isabella Castellano, Eleonora Marinelli, Simona Osella-Abate, Anna Sapino, Paola Cassoni, Mauro Papotti

**Affiliations:** 1 Department of Oncology, Pathology Unit, University of Turin, Via Santena 7, 10126 Turin, Italy; 2 Department of Medical Sciences, Pathology Unit, University of Turin, Via Santena 7, 10126 Turin, Italy; 3 Pathology Division, Candiolo Cancer Institute, FPO-IRCCS, 10060 Candiolo, Italy

**Keywords:** INSM1, Neuroendocrine differentiation, Breast carcinoma, Histotype

## Abstract

According to the 2019 WHO classification of breast tumors, neuroendocrine neoplasms (NENs) are classified into well-differentiated NE tumors (NET) and poorly differentiated NE carcinomas (NEC), while other breast cancers (BCs) of special and no special type with neuroendocrine (NE) features are not incorporated in this scheme anymore. We aimed to assess whether INSM1, a novel NE marker, could have a role in breast NEN subtyping. We selected 63 BCs operated from 2003 to 2018, classified as BCs with NE features, with available clinico-pathological data. Following 2019 WHO criteria, this cohort was reclassified into 37 NETs/NECs, the remaining 26 tumors representing solid-papillary (7), mucinous (7), and mixed type (12) carcinomas with NE differentiation. Chromogranin A (CGA) and synaptophysin (SYN) immunostains were reviewed, and INSM1 was tested by immunohistochemistry. Thirty CGA- and SYN-negative no special type BCs served as negative control. INSM1 was expressed in 52/63 cases of the whole cohort (82.54%). INSM1 positive and negative cases had no significantly different clinico-pathological characteristics. INSM1 expression was not significantly different between the newly reclassified NET/NEC group and other BCs with NE features. No immunoexpression was observed in control BCs. The sensitivity and specificity of INSM1 for the NE phenotype was 82.5% and 100%, respectively, compared to 61.9% and 100% for CGA, and 95.2 and 100% for SYN. In conclusion, INSM1 is as accurate as traditional NE biomarkers to identify NE differentiation in BC. In analogy to standard NE markers, INSM1 could not distinguish NET and NEC from the other BC histotypes with NE differentiation.

**Supplementary Information:**

The online version contains supplementary material available at 10.1007/s12022-021-09682-1.

## Introduction

The definition of neuroendocrine neoplasms (NEN) of the breast is hampered by continuous efforts to identify precise diagnostic criteria, as reflected by the different classification systems proposed by the WHO schemes in 2003 [1], 2012 [2], and 2019 [3].

In particular, the last WHO edition [3] proposes to classify NEN of the breast into NE tumors (NET) and NE carcinomas (NEC), in analogy to NEN originating from the gastroenteropancreatic tract and lung [4].

NET and NEC in the breast, as defined by the latest WHO classification, are characterized by well-differentiated and poorly differentiated neuroendocrine morphology, respectively, and they both show ultrastructural and immunohistochemical (IHC) features of neuroendocrine differentiation, supported by the presence of neurosecretory granules and a diffuse immunoreactivity for NE markers. Specifically, NETs are well or intermediate differentiated tumors, while NECs are poorly differentiated BC. BCs with NE features, such as solid papillary carcinoma, pure mucinous carcinoma (representing special BC histotypes), and tumors with mixed histology, have been excluded from the current WHO classification of breast NEN [3].

A novel marker called insulinoma-associated protein 1 (INSM1) has been identified in insulinoma tissue [5] and subsequently detected in different NE human cells and tumors. INSM1 is a zinc-finger transcription factor that through interactions with ASH1 and BRN2 [6, 7] favors the expression of well-established NE markers chromogranin A (CGA), synaptophysin (SYN), and CD56 [8, 9], representing key regulators of NE differentiation [6, 10, 11]. In healthy tissues, the nuclear expression of INSM1 is limited to NE cells of pancreatic islets, adrenal medulla, gastro-intestinal, and bronchopulmonary tract [12–14]. Moreover, INSM1 is strongly expressed in most NE tumors, with a specific nuclear staining [15]. However, to the best of our knowledge, only few BC with NE features were investigated for INSM1 expression [16–18].

Considering this background, aims of this study are (i) to test INSM1 specificity and sensitivity for the NE phenotype in BC and (ii) to assess whether INSM1 expression may differentiate the novel NET/NEC categories from the other BC with NE features.

## Material and Methods

### Case Selection

From the pathology files of the Città della Salute e della Scienza University Hospital in Turin, key words such as “breast,” “carcinoma,” “neoplasm,” “infiltrative,” and “neuroendocrine” were searched to select a series of 63 BC with NE features, operated from 2003 to 2018, with sufficient residual material for IHC investigations. A series of 30 invasive BC of no special type (NST) with no expression of CGA and SYN served as the control group.

For each case, clinical-pathological data such as age, site of lesion, type of surgery, type of therapy, and follow up data were obtained from clinical charts. In addition, information regarding tumor size, lymph nodal status, histological grade, vascular invasion, estrogen (ER), progesterone (PgR), human epidermal growth factor receptor 2 (HER2) receptor status, and Ki67 proliferation index were retrieved from pathological reports. Both ER and PgR were considered as positive if more than 1% of tumor cells had a nuclear immunostaining [19]. HER2 status was classified as negative (score 0, 1+, and 2+ not amplified) or positive (when scored 3+ by IHC or HER2 amplified by FISH) according to the recommended guidelines for invasive carcinoma [20]. CGA and SYN expressions were recorded, as well.

Surrogate molecular profile was obtained according to the recommendations of St. Gallen 2013 [21].

**Fig. 1:**
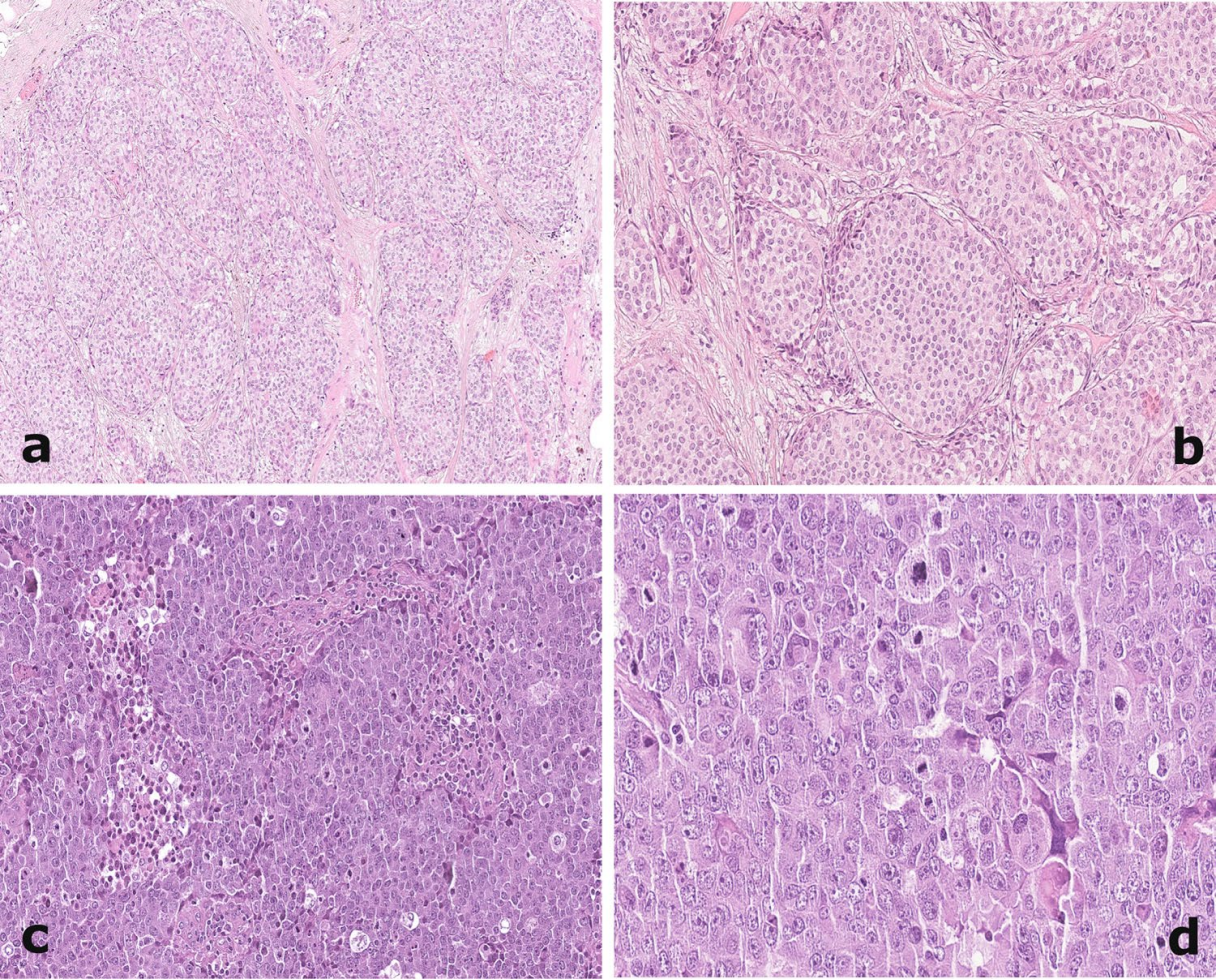
A case of neuroendocrine breast tumor (**a**/**b**, 100×/200×) showing typical solid nest growth separated by fibrovascular stroma. A case of neuroendocrine breast carcinoma (**c**/**d**, 200×/400×) displaying high number of mitotic figures, pleomorphic nuclei, and foci of necrosis

Each case was reviewed by the three of us (JM, IC, MP) and reclassified strictly following the criteria of the last 2019 WHO edition [3]. Specifically, NET diagnosis was referred to neoplasms with typical solid nests or trabeculae of spindle/polygonal/plasmacytoid cells, separated by fibrovascular stroma (Fig. 1a/b). The diagnosis of NEC was restricted to high-grade tumors, morphologically resembling pulmonary high-grade NE carcinomas. NEC were composed either of small cells with extensive necrosis, uniform small dark hyperchromatic cells with high nuclear/cytoplasm ratio, or large cells with evident cytoplasm and highly pleomorphic nuclei (Fig. 1c/d). At least one extensive positivity of traditional NE marker (CGA and/or SYN) was required to confirm the morphological diagnosis.

The study was approved by the Research Ethics Committee for Human Biospecimen Utilization (Department of Medical Sciences—ChBU) of the University of Turin (no. 5/2020). Written consent was not required considering the retrospective nature of the study. The study was conducted in accordance with The Code of Ethics of the World Medical Association (Declaration of Helsinki). All cases were de-identified, and all clinical-pathological data were accessed anonymously.

### Immunohistochemistry

All cases were stained for INSM1 (clone A8, Santa Cruz Biotechnology, Santa Cruz, CA, diluted 1:100) using an automated immunostainer platform (BenchMark AutoStainer, Ventana Medical Systems, Tucson, AZ, USA). The intensity (weak to strong) and the percentage of positive tumor cell nuclei were recorded. The cut off for a positive reaction was set at 5% of tumor cells, as previously reported [22]. Pancreatic tissue, representing appropriate positive (Langerhans islets) and negative (acinar cells) controls, was included in each IHC run.

When missing from the file, CGA (clone LK2H10, Ventana, prediluted) or SYN (clone SP11, Ventana, prediluted) were tested using the BenchMark AutoStainer. Both markers were classified as (i) diffusely positive if present in more than 50% of tumor cells, (ii) focally positive if present in < 50% of tumor cells, and (iii) absent if no staining was observed.

### Statistical Analysis

Statistical analyses were carried out using Stata 15.0 software (StataCorp, College Station, TX, USA). The differences in the distribution of the variables evaluated based on clinical-pathological parameters were analyzed using parametric and non-parametric tests (Student’s *t* test, Pearson’s chi-square test and Bonferroni’s correction, Wilcoxon’s rank test).

Sensitivity, specificity, positive predictive value (PPV), and negative predictive value (NPV) were calculated, as previously described [23].

Time to relapse (relapse-free interval—RFI) was assessed from the date of diagnosis to the date of relapse or the date of the last checkup. Overall survival (OS) was assessed from the date of diagnosis to the date of death from any cause or to the date of the last checkup. All dead patients were considered as events.

Survival analysis was determined by the Kaplan–Meier curves, and Mantel log-rank test was used to compare statistical differences.

## Results

Upon revision, 37 cases were recorded as NENs, namely, 9 NET G1, 20 NET G2, and 8 NEC, the latter group consisting of 6 large-cell and 2 small-cell neuroendocrine carcinomas. The remaining 26 tumors despite more or less extensive expression on NE markers did not meet the morphological criteria recommended by 2019 WHO scheme and were referred as “BC with NE differentiation.” This group included solid papillary (7 cases), mucinous (7 cases), and mixed type (12 cases) carcinomas (having a NE morphology in < 10% of the tumor area).

### NE Marker Expression in the Whole Series

**Fig. 2:**
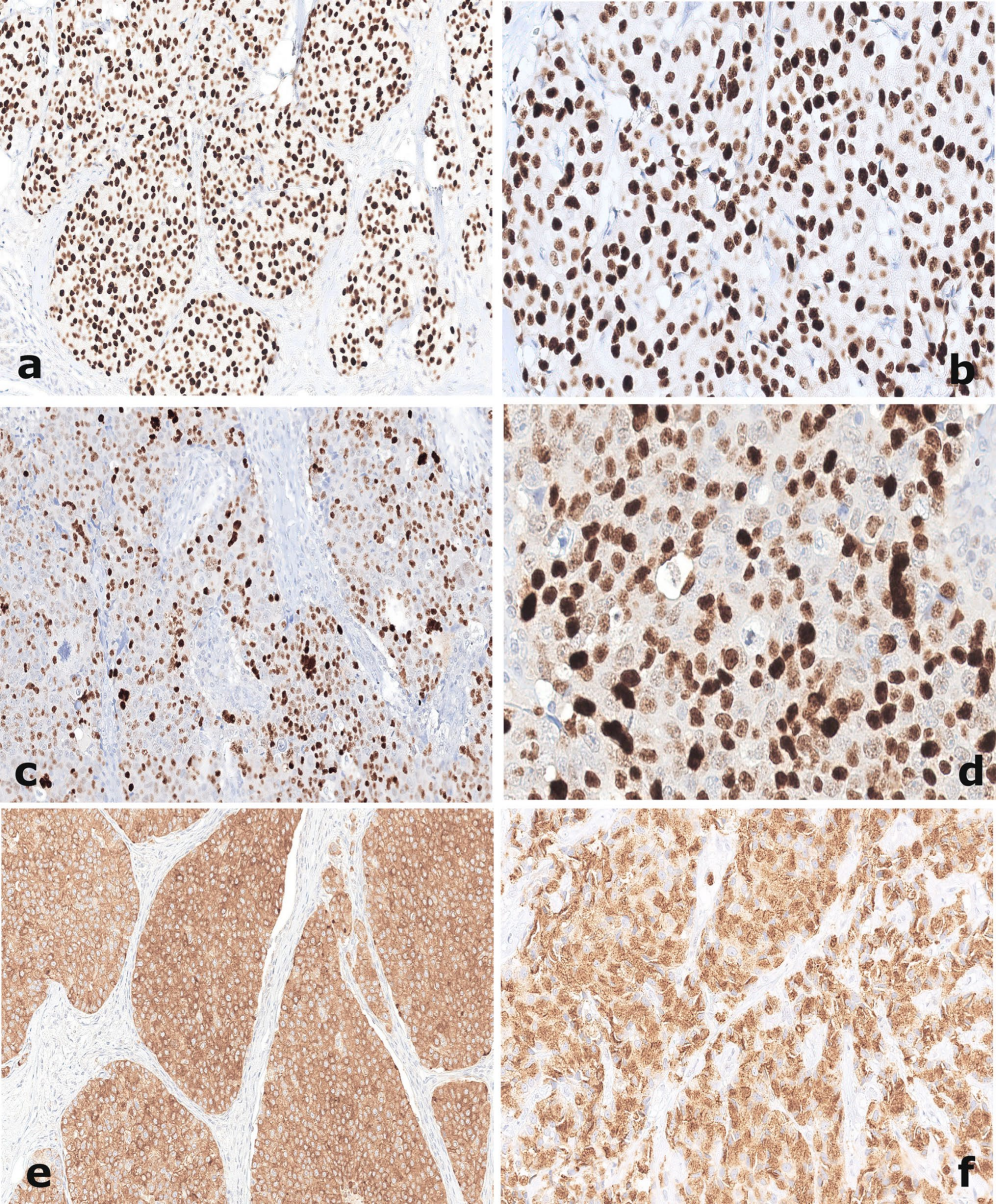
Strong and diffuse nuclear INSM1 immunoreactivity in a case of neuroendocrine breast tumor (**a**/**b**, 200×/400×) and in a case of neuroendocrine breast carcinoma (**c**/**d**, 200×/400×). Diffuse immunoexpression of traditional neuroendocrine markers, Synaptophysin (**e**, 200×) and Chromogranin A (**f**, 200×) demonstrated in a case of neuroendocrine breast tumor

INSM1 was expressed in 52/63 (82.5%) of the whole cohort (Fig. 2a–d). INSM1 negative and positive cases did not demonstrate statistically significant differences in clinico-pathological characteristics, nor regarding the surrogate molecular profile status, as shown in Table 1. SYN and CGA were positive in 60/63 (95.2%) and 39/63 (61.9%) cases, respectively (Table 1; Fig. 2e/f).

**Table 1:** Clinico-pathological features of 63 neuroendocrine differentiated breast cancers according to INSM1 expression

Features		INSM1 NEGATIVE #11	INSM1 POSITIVE #52	*P* value	Total (63)
Age (years)	Median (interval)	72 (56–93)	75 (45–86)	0.545	74 (45–93)
Histological grade	1	4	13	0.615	17
	2	6	29		35
	3	1	10		11
Vascular invasion	No	7	28	0.553	35
	Yes	4	24		28
pT	1	7	31	0.824	38
	2	4	17		21
	3	0	2		2
	4	0	2		2
pN	0	7	38	0.340	45
	1	4	10		14
	2	0	4		4
ER	Median (interval)	95 (80–99)	99 (10–100)	0.121	99 (10–100)
PgR	Median (interval)	18 (0–98)	80 (0–100)	0.702	78 (0–100)
PgR	< 20	4	11	0.282	15
	≥ 20	7	41		48
HER2	0	10	28	0.067	38
	1+	1	14		15
	*2+	0	10		10
Surrogate molecular profile	Luminal A	4	29	0.242	33
	Luminal B	7	23		30
Ki67 index	< 20	7	30	0.716	37
	≥ 20	4	22		26
Ki67 index	Median (interval)	18 (4–43)	17 (1–80)	0.886	18 (1–80)
CGA	Negative	5	19	0.580	24
	Focal/diffuse positivity	6	33		39
SYN	Negative	0	3	0.414	3
	Focal/diffuse positivity	11	49		60
Surgery	Conservative	5	31	0.389	36
	Mastectomy	6	21		27
Recurrences (missing 9)	No	9	39	0.901	48
	Yes	1	5		6
Died of disease (missing 3)	No	7	39	0.258	46
	Yes	4	10		14

*ER* estrogen receptor, *PgR* progesterone receptor, *CGA* Chromogranin A, *SYN* Synaptophysin

^*^All cases that were assessed as 2+ underwent FISH analyses that resulted negative for HER2 gene status

**Fig. 3:**
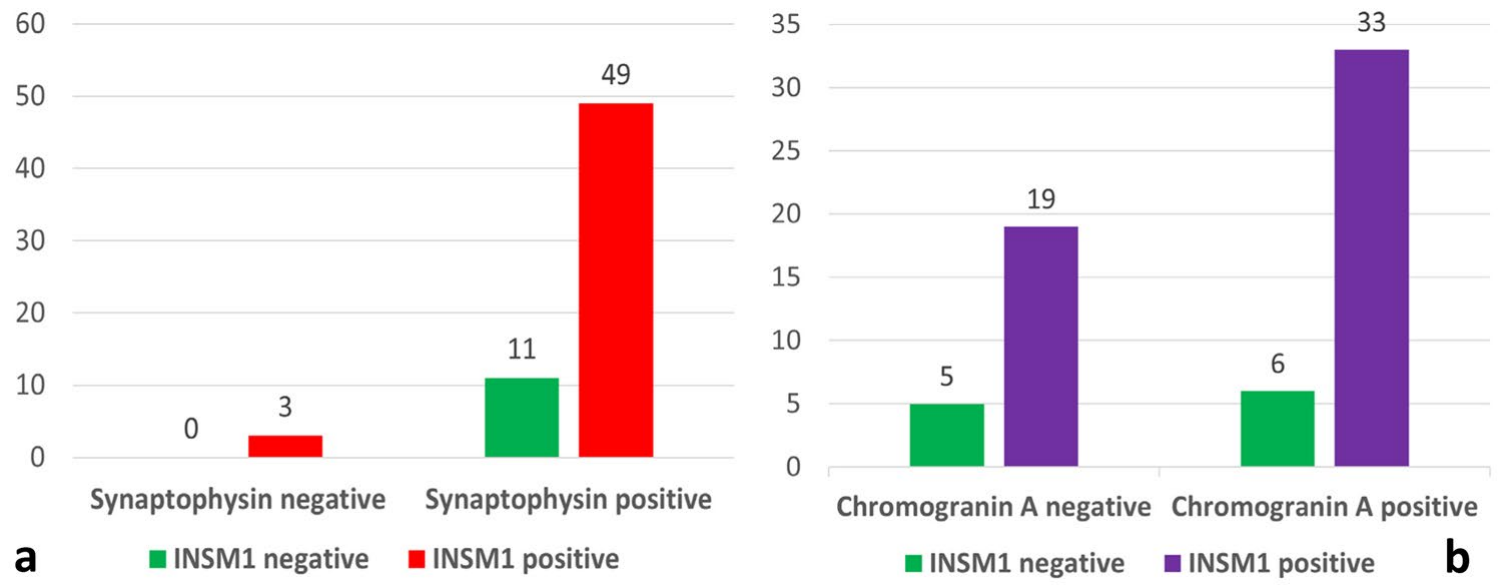
A chart showing INSM1 and Synaptophysin discordant expression in 14/63 cases **a** and INSM1 and Chromogranin A discordant expression in 25/63 cases **b**

INSM1 and SYN expression was discordant in 14/63 cases (22%) (11 INSM1 negative and SYN positive and 3 INSM1 positive and SYN negative) (Fig. 3a). INSM1 and CGA expression was discordant in 25/63 (39.7%) tumors (6 INSM1 negative and CGA positive and 19 INSM1 positive and CGA negative), indicating that INSM1 identifies few SYN-negative cases and a relevant fraction of CGA-negative tumors (Fig. 3b). INSM1 expression was not detected in any of the 30 NST control BC. Clinico-pathological characteristics of these cases are reported in Supplementary Table 1. Hence, INSM1 showed 82.54% (70.90–90.95, *95%* CI) sensitivity and 100% (88.43%-100.0%, *95%* CI), specificity, with PPV 100% and NPP 73.17% (61.45–82.35%, 95% CI). In this series, CGA sensitivity was 61.90% (48.8–73.85, 95% CI), specificity 100% (88.43–100%, 95% CI), PPV 100%, and NPV 55.5% (47.71–63.13, 95% CI); SYN sensitivity was 95.24% (86.71–99.01, 95% CI), specificity 100% (88.43–100%, 95% CI), PPV 100%, and NPV 90.91% (76.82–96.79, 95% CI).

### Comparison of NE Marker Expression in NET/NEC and BC with NE Differentiation, According to 2019 WHO Classification

No significant differences were observed among the new NET/NEC categories (37 cases) and BC with NE differentiation cancers (26 cases) regarding clinico-pathological features and CGA expression (Table 2). Conversely, SYN immunoreactivity was preferentially observed in NENs (100%) compared with BC with NE differentiation (88.5%, *P* = 0.034).

**Table 2:** Clinical pathological features of whole case series according to 2019 WHO classification

		NET/NEC (37)	Other NE differentiated BC (26)	*P* value	Total
Age (years)	Median (interval)	73 (45–93)	75 (46–86)	0.881	74 (45–85)
Histological grade	1	9	9	0.560	17
	2	20	15		35
	3	8	3		11
Vascular invasion	No	20	15	0.775	35
	Yes	17	11		28
pT	1	24	14	0.416	38
	2	10	11		21
	3	1	1		2
	4	2	0		2
pN	0	26	19	0.791	45
	1	8	6		14
	2	3	1		4
ER	Median (interval)	99 (10–100)	95 (15–100)	0.452	99 (10–100)
PgR	Median (interval)	65 (0–100)	80 (0–99)	0.584	60 (0–100)
HER2	0	23	15	0.829	38
	1+	9	6		15
	*2+	5	5		10
Surrogate molecular profile	Luminal A	18	15	0.479	33
	Luminal B	19	11		30
Ki67 index	< 20	21	16	0.704	37
	≥ 20	16	10		26
Ki67 index	Median (interval)	18 (1–80)	16 (2–45)	0.740	18 (1–80)
CGA	Negative	13	11	0.564	24
	Focal/diffuse positivity	24	15		39
SYN	Negative	0	3	0.034	3
	Focal/diffuse positivity	37	23		60
INSM1	Negative	9	2	0.087	11
	Positive	28	24		52
Surgery	Conservative	18	18	0.722	36
	Mastectomy	19	8		27
Type of therapy (13 missing)	HT	25	18	0.506	43
	HT and/or CT	5	2		7
Recurrences (9 missing)	No	28	20	0.236	48
	Yes	5	1		6
Died of disease (3 missing)	No	25	21	0.105	46
	Yes	11	3		14

*NE* neuroendocrine, *BC* breast cancer, *ER* estrogen receptor, *PgR* progesterone receptor, *CGA* Chromogranin A, *SYN* Synaptophysin, *HT* hormonal therapy, *CT* chemotherapy

^*^All cases that were assessed as 2+ underwent FISH analyses that resulted negative for HER2 gene status

INSM1 was expressed in 28/37 (75.7%) NET/NEC and 24/26 (92.3%) other BC with NE features (*P* not significant) (Table 2). The intensity and percentage of INSM1 nuclear reactivity were similar in the tumor cell population of NETs and of NECs (Table 3). The extent of biomarker immunoreactivity is summarized in Supplementary Table 2.

**Table 3:** Median percentage distribution of INSM1 in 37 pure NET/NEC cases, according to WHO 2019

	INSM1 positive cases /total cases	Extent of INSM1 expression (Median %)	Range
NET	21/29 (72%)	60	5–100
NEC	7/8 (78%)	70	10–100

Furthermore, no significant clinico-pathological or prognostic differences were detected between INSM1 positive and negative cases in the NET/NEC group (data not shown).

## Discussion

INSM1, a transcription factor expressed during development and maturation of NE cells, has been demonstrated a highly sensitive and specific marker of NE differentiation in pulmonary [24, 25] and gastroenteropancreatic NENs [26, 27]. Its nuclear expression was easily recognizable in small biopsies and cytological specimens with scarce material, contributing to diagnostic accuracy in challenging cases [28, 29]. Although extensively studied, to date, only few papers [16–18, 22] reported INSM1 immunoreactivity in BC. The so far reported cases were selected with different criteria, being described as BC with NE differentiation and, when indicated, they mostly included mucinous, solid papillary, or mixed carcinoma variants, which are not included in the currently proposed WHO categories of breast NEN [3]. In addition, two studies were performed only on tissue microarray cores, which may not allow reliable comparisons among the various series.

In our cases, INSM1 expression was observed in approximately 80% of BC with NE features, with a high sensitivity and specificity, representing an optimal adjunct in the determination of NE differentiation in BC. However, its expression did not correlate with clinical-pathological characteristics.

In line with other studies, our data demonstrated that INSM1 is more sensitive than CGA but, unlike in other organs, less sensitive than SYN to reveal NE differentiation in BC. Its specificity overlapped that of CGA and SYN [30].

In a previous study from our group [8], another NE marker, the transcription factor hASH1, generally expressed by high-grade NEC of various organs, was investigated in a BC series with NE differentiation (including 17 cases also analyzed in the present study). In the NE cell population, hASH1 expression was found in 63% and 38% of cases with an extensive (> 50%) or focal (< 50%) reactivity, respectively. The concordance between hASH1 and INSM1 reactivity was low (41.2%), as also observed in pulmonary and extrapulmonary NEC. This is probably related to the fact that INSM1 is a target of hASH1 (i.e., *ASCL1* gene), but also of the *NEUROD1* gene. Thus, in pulmonary and extrapulmonary sites (probably including breast, based on the present findings), INSM1 expression correlates with either one or the other gene, being mutually exclusive and involved in the regulation of different pathways [31–33].

Reviewing all tumors according to the latest WHO edition of BC classification, only 37 (58.73%) cases out of the 63, originally reported as BC with NE differentiation, have been reclassified as NET/NEC. The remaining 26 cases did not meet the novel diagnostic criteria, representing cases of solid-papillary, mucinous, or mixed carcinomas. However, no significant differences were observed in terms of INSM1 expression in the novel NEN categories, compared to the other BC with NE features. Specifically, INSM1 expression was found in 75.7% of NET/NEC and in 92.3% of the other histotypes, as expected, being a general marker of NE differentiation.

The latest WHO proposal [3] attempted to make a uniform classification concept, applicable to all human NE tumors, as recommended by a joint International Agency for Research on Cancer (IARC) and WHO group of experts [4]. However, in the BC field, considering that other histotypes may show NE differentiation, traditional NE biomarkers, including INSM1, do not seem to be effective in distinguishing the newly proposed NET/NEC categories of the breast [3, 4]. In addition, the median expression of INSM1 was similar in NET and NEC cases. These data reinforce its utility in identifying NE differentiation, independently from the intrinsic biological aggressiveness of the NEN subtypes, as described in other organs [25]. In addition, pure breast NENs are probably very rare, being more common the mixed forms, in which an exocrine component co-exists with a NE differentiated cell population. This renders challenging the possibility of perfectly fitting the rigid scheme of NEN classification into NET and NEC, as recently discussed in the review by Uccella and co-workers [34]. In fact, they questioned the attempt to align the classification criteria of NE BC with those of other organs, in the absence of a univocally recognizable morphology and of a clinical behavior different from that of non-NE conventional BC.

The currently described extensive INSM1 expression in BC with NE differentiation confirms that divergent differentiation driven by known NE-related transcription factors may occur in BC, resulting in a spectrum of tumors, which in part overlap NENs of other organs and in part represent combined/mixed neuroendocrine-exocrine BCs. Further investigations on the significance of NE features in BC are warranted.

In conclusion, our results showed that INSM1 is an accurate NE biomarker that can be employed, together with CGA and SYN, to confirm NE features in BC. However, all these markers recognize a phenotype and not a precise NE entity and cannot be used to distinguish NET/NEC from other breast cancer types with NE differentiation.

## Supplementary Information

Below is the link to the electronic supplementary material.

Supplementary file1 (DOCX 18 KB)

Supplementary file2 (DOCX 15 KB)
