# Supplementary material for: INSM1 Expression in Breast Neoplasms with Neuroedocrine Features
Source: Endocr Pathol. 2021 May 19;32(4):452–60. doi: 10.1007/s12022-021-09682-1 (PMC8608773; doi:10.1007/s12022-021-09682-1)
Supplement: Supplementary file 1 — Supplementary file1 (DOCX 18 KB) [file 12022_2021_9682_MOESM1_ESM.docx]

**Supplementary Table 1.** Clinico-pathological characteristics of 30 non-neuroendocrine breast cancers of the control series.

| Features |  | #30 |
| --- | --- | --- |
| Age (years) | Median (interval) | 57 (36-81) |
| Histological grade | 1 | 4 |
|  | 2 | 20 |
|  | 3 | 6 |
| Vascular invasion | no | 15 |
|  | yes | 15 |
| pT | 1 | 16 |
|  | 2 | 13 |
|  | 3 | 0 |
|  | 4 | 1 |
| pN | 0 | 18 |
|  | 1 | 9 |
|  | 2 | 3 |
| ER | Median (interval) | 96 (10 - 100) |
| PgR | Median (interval) | 90 (10 -100) |
| HER2 | 0 | 11 |
|  | 1+ | 11 |
|  | *2+ | 5 |
|  | 3+ | 3 |
| Ki67 index | Median (interval) | 15 (2 - 90) |
| Surrogate molecular profile | Luminal A | 20 |
|  | Luminal B | 10 |
| Surgery | conservative | 21 |
|  | mastectomy | 9 |
| SYN/CGA/INSM1 | negative | 30 |
|  | positive | 0 |

Abbreviations: ER: estrogen receptor, PgR: progesterone receptor, CGA: Chromogranin A, SYN: Synaptophysin.

*All cases that were assessed as 2+ underwent FISH analyses that resulted negative for HER2 gene status.
