# Supplementary material for: INSM1 Expression in Breast Neoplasms with Neuroedocrine Features
Source: Endocr Pathol. 2021 May 19;32(4):452–60. doi: 10.1007/s12022-021-09682-1 (PMC8608773; doi:10.1007/s12022-021-09682-1)
Supplement: Supplementary file 2 — Supplementary file2 (DOCX 15 KB) [file 12022_2021_9682_MOESM2_ESM.docx]

| Immunohistochemical marker |  | NET#29 | NEC#8 | BC with NE differentiation #26 | *P* value |
| --- | --- | --- | --- | --- | --- |
| SYN | negative | 0 | 0 | 1 | 0.345 |
|  | diffuse | 29 | 8 | 23 |  |
|  | focal | 0 | 0 | 2 |  |
| CGA | negative | 11 | 2 | 11 | 0.549 |
|  | diffuse | 11 | 3 | 12 |  |
|  | focal | 7 | 3 | 3 |  |
| INSM1 | negative | 8 | 1 | 2 | 0.141 |
|  | positive | 21 | 7 | 24 |  |
| INSM1 intensity | negative | 8 | 1 | 2 | 0.361 |
|  | 1+ | 1 | 0 | 3 |  |
|  | 2+ | 3 | 2 | 3 |  |
|  | 3+ | 17 | 5 | 18 |  |
| INSM1 % | Median (interval) | 40 (0-100) | 70 (0 – 100) | 55 (0 – 100) | 0.736 |
| Ki67 | <20% | 21 | 0 | 16 | <0.001 |
|  | ≥20% | 8 | 8 | 10 |  |

**Supplementary Table 2.** Extent of biomarker reactivity, including Synaptophysin, Chromogranin A, INSM1 and Ki67 in the whole series (#63).

Abbreviations: CGA: Chromogranin A, SYN: Synaptophysin.
